# Perceived discrimination and health outcomes among middle-aged and older adults in India: results of a national survey in 2017–2018

**DOI:** 10.1186/s12877-021-02508-z

**Published:** 2021-10-18

**Authors:** Supa Pengpid, Karl Peltzer

**Affiliations:** 1 ASEAN Institute for Health Development, Mahidol University, Salaya, Phutthamonthon, Nakhon Pathom, Thailand; 2 Department of Research Administration and Development, University of Limpopo, Turfloop, Polokwane, South Africa; 3 Department of Psychology, College of Medical and Health Science, Asia University, Taichung, Taiwan

**Keywords:** Perceived discrimination, Physical health, Mental health, Health risk behaviour middle-aged, Older adults, India

## Abstract

**Background:**

The study aimed to estimate the associations between perceived discrimination and poor physical health, poor mental health, and health risk behaviours in middle-aged and older adults in a national population survey in India.

**Methods:**

The sample included 72,262 middle-aged and older adults from a cross-sectional national community dwelling survey in India in 2017–2018.

**Results:**

The prevalence of moderate (1–2 types) perceived discrimination was 10.7%, and high (3–6 types) perceived discrimination was 6.6%. In the final adjusted logistic, linear or Poisson regression analyses, moderate and/or high perceived discrimination was significantly positively associated with poor mental health (low life satisfaction, poor cognitive functioning, insomnia symptoms, and depressive symptoms), poor physical health (pain conditions count, and functional limitations), and health risk behaviours (heavy episodic drinking and physical inactivity).

**Conclusion:**

Perceived discrimination is associated with poor mental health, poor physical health, and health risk behaviour, emphasising the need to consider perceived discrimination in various physical and mental health contexts.

## Background

Perceived discrimination can be defined as “a behavioural manifestation of a negative attitude, judgment, or unfair treatment toward members of a group”, such as age, gender, race and ethnicity, religion, caste, weight, physical disability, physical appearance, and financial status, and can be characterised as a form of stress [1]. There are two different types of perceived discrimination, one is referring to major lifetime experiences including singular incidents in society such as the housing or labour market, and the second type is everyday perceived discrimination including daily interpersonal hassles and insults encompassing chronic psychosocial stressors [2]. There are several pathways on how perceived discrimination affects health [3]. Following ecosocial theory “lived realities of.

discrimination as an exploitative and oppressive societal phenomenon operating.

at multiple levels and involving myriad pathways across both the life course and historical.

generations” need to be considered [4]. Routine discrimination may become a chronic stressor weaking a person’s protective and self-control resources and thereby increasing one’s vulnerability to detrimental mental, physical, and behavioural health outcomes [1]. Perceived discrimination has been associated with negative mental health outcomes, such as depressive symptoms [1, 5, 6], psychiatric distress, poor general well-being [1], poor mental health [7], poor sleep [8, 9], poorer cognitive functioning [10], poorer physical health [1], such as poorer self-rated health status [4, 6, 11], poor self-rated oral health status [4], chronic illness [6], cardiovascular conditions [4, 12], respiratory conditions [4, 12], pain conditions [4, 12], poorer physical functioning or functional limitations [6, 10], multimorbidity [4], and unhealthy behaviours [1], such as smoking [1, 9] and alcohol use [1], and increased exposure to the healthcare setting [11].

Most studies investigating the association between perceived discrimination and health outcomes have been conducted in high-income countries, such as in USA, and there has been a lack of studies in low- and middle-income countries, such as in India. Some studies found a high prevalence of everyday perceived discrimination, e.g., in a study among older adults (≥50 years) in Brazil, the prevalence everyday perceived discrimination was 16.8% [13], and among pregnant women in rural Gujarat in India, 67.6% reported at least one type of everyday perceived discrimination [14]. Gender discrimination has been found associated with poor mental health among women in Bihar, Jharkhand, and Maharashtra in India [15], and everyday perceived discrimination was associated with poorer health status in China [16]. Middle-aged and older adults may be at higher risk of being discriminated against due to their decreasing social and financial status [17].

The study aimed to estimate the associations between perceived discrimination and poor physical health, poor mental health, and health risk behaviours in middle-aged and older adults in a national population survey in India. Higher everyday perceived discrimination scores were hypothesized to relate to higher poor physical health, poor mental health, and health risk behaviours.

## Methods

### Sample and procedures

National cross-sectional data from the Longitudinal Aging Study in India (LASI) Wave 1, 2017–2018 were analyzed (individual response rate 87%) [17]. Face-to-face interviews, physical measurement and biomarker data were collected by male and female trained research teams from individuals aged 45 and above and their female spouses, regardless of age, in a household survey from 35 states and union territories of India (excluding Sikkim). Field teams input responses directly into a laptop computer. Details of the sampling strategy have been described elsewhere [17]. Briefly, “LASI adopted a multistage stratified area probability cluster sampling design to arrive at the eventual units of observation: older adults aged 45 and above and their spouses irrespective of age. India is a union comprising 30 states and 6 union territories. Within each state, LASI Wave 1 adopted a three-stage sampling design in rural areas and a four-stage sampling design in urban areas. In each state/union territories, the first stage involved the selection of Primary Sampling Units (PSUs), that is, subdistricts (Tehsils/Talukas), and the second stage involved the selection of villages in rural areas and wards in urban areas in the selected PSUs. In rural areas, households were selected from selected villages in the third stage. However, sampling in urban areas involved an additional stage. Specifically, in the third stage, one Census Enumeration Block (CEB) was randomly selected in each urban area. In the fourth stage, households were selected from this CEB. Goal was to select a representative sample in each stage of sample selection.” [17]. Persons 65 years and older were oversampled by including households with at least one person aged 65 and above to increase the sample size for the elderly age 65 [17]. The Indian Council of Medical Research (ICMR) Ethics Committee approved the study and written/oral informed consent was attained from participants [17].

### Measures

#### Outcome variables

##### Mental health

*Life satisfaction* was assessed with the item, “Please, think about your life as a whole. How satisfied are you with it? Are you completely satisfied, very satisfied, somewhat satisfied, not very satisfied, or not at all satisfied?” This item was adapted from the Health and Retirement Study [17], and “is a validated single-item measure of life satisfaction that has been widely used in prior research and correlates strongly with richer, multiple-item life satisfaction measures” [18, 19]. Responses were grouped into 1 (low) = not very satisfied or not at all satisfied, and 0 = somewhat satisfied or very satisfied or completely satisfied.

*Cognitive functioning* was assessed with tests for immediate and delayed word recall, serial 7 s, and orientation based on the Mini-Mental State Exam [20]. A composite score of 0–32 was computed with a higher score representing better cognitive functioning.

*Insomnia symptoms* were assessed with four questions adapted from the Jenkins Sleep Scale (JSS-4) [21]: “How often do you have trouble falling asleep?” 2) “How often do you have trouble with waking up during the night?” 3) “How often do you have trouble with waking up too early and not being able to fall asleep again?” 4) “How often did you feel unrested during the day, no matter how many hours of sleep you had?” Response options were “never, rarely (1-2 nights per week), occasionally (3-4 nights per week), and frequently (5 or more nights per week)”(item 4 was reverse coded). Insomnia problems were “coded as ‘frequently’ for any of the four symptoms as 1 and 0 for other responses. Participants who scored 1 on any of the four symptoms were considered to have insomnia symptoms.” [22]. The “JSS-4 proved excellent reliability and it demonstrated good construct validity.” [23]. Internal consistency of the JSS-4 was 0.80 in this study.

*Depressive symptoms* were measured with the Centre for Epidemiological Studies Depression Scale (CES-D-10) [24]. The overall scores ranged from zero to 10 and scores of four or more were classified as having depressive symptoms [25]. (Cronbach α was 0.79 in this study).

*Pain conditions* were assessed with four items. “Have you had any of the following persistent or troublesome problems in past two years?” Back pain or problem, Pain or Stiffness in joints, Pain or Stiffness in joints (Yes/No) and “In the last 12 months, have you ever been diagnosed with or suffered from painful teeth” (Yes/No) [17]. The four pain conditions were summed and used as a binary measure (1 = any pain condition and 0 = no pain condition) in the descriptive table and as a count measure (number of pain conditions) in the Poisson regression model.

##### Physical health

*Cardiovascular conditions: Hypertension or raised blood pressure* (BP) was defined as “systolic BP ≥140 mm Hg and/or diastolic BP ≥90 mm Hg (based on the last two averaged of three readings) or where the participant is currently on antihypertensive medication.” [26]. Health care provider diagnosed conditions included 1) Chronic heart diseases such as coronary heart disease (heart attack or Myocardial Infarction), congestive heart failure, or other chronic heart problems, and 2) Stroke [17]. *Angina* was assessed with the “World Health Organization’s Rose angina questionnaire” [27], and defined on the basis of “discomfort at walking uphill or hurrying, or at an ordinary pace on level ground. Furthermore, the pain should be located at the sternum or in the left chest and arm, causing the patient to stop or slow down, and the pain should resolve within 10 minutes when the patient stops or slows down.” [28]. The four cardiovascular conditions were summed and used as a binary measure (1 = any cardiovascular condition and 0 = no cardiovascular condition) in the descriptive table and as a count measure (number of cardiovascular conditions) in the Poisson regression model. *Chronic lung disease* such as “asthma, chronic obstructive pulmonary disease/chronic bronchitis, or other chronic lung problems” was assessed by self-reported health care provider diagnosis (Yes/No) [17]. *Functional limitations* were assessed with Activities *of Daily Living* (ADL) (6 items) and Instrumental Activities of Daily Living (IADL) (7 items) (Yes, No) [29, 30]. Cronbach alpha was 0.87 for ADL and 0.90 for IADL in this study. Responses were dichotomized into 0–1 and ≥ 2 ADL and IADL items.

##### Health risk behaviours

*Current tobacco smoking* was sourced from the item, “Do you currently smoke any tobacco products (cigarettes, bidis, cigars, hookah, cheroot, etc.)?” (Yes, No) [17]. *Heavy episodic alcohol use* was assessed with the question, “In the last 3 months, how frequently on average, have you had at least 5 or more drinks on one occasion?” [17] and defined as “one to three days per month, one to four days per week, five or more days per week, or daily.” *Physical inactivity* was defined as hardly ever or never engaging in vigorous or moderate physical activity [17].

#### Exposure variable

*Discrimination experiences* were assessed with the six-item Everyday Discrimination Scale (EDS) (Short version) [6]. The EDS measures subjective experiences of discrimination, defined as “the belief that one has experienced unfair treatment by individuals and social institutions, and that this treatment was based on personal characteristics such as race, gender, or weight” [31]. The wording of each item, e.g., “treated with less courtesy or respect,” are shown in Table 2). Response options ranged from 1 = “never” to 6 = “almost every day” were dichotomised to ‘never’ = 0 and ‘ever’ (collapsing those reporting ‘less than once a year’ or greater into one category) = 1, summed with total scores from 0 to 6, and trichotomized into 0 = 1 no, 1–2 = 2 moderate and 3–6 = 3 high discrimination; Cronbach’s alpha for the EDS in this study was 0.86. Participants that responded affirmative to any discrimination question were asked a follow-up question inquiring into the potential reasons for discrimination. Response options were “age, gender, religion, caste, weight, physical disability, other aspects of physical appearance, financial status, and others.” [6].

*Covariates* consisted of education (none and ≥ 1 years), age, sex (male, female), marital status (currently married vs. widowed/divorced/separated/deserted/live-in relationship/never married), caste (Scheduled tribes, scheduled castes, other backward classes, and one of these) urban and rural residence [17]. Scheduled tribes, scheduled castes, and other backward classes have been historically disadvantaged due to various socio-economic factors like wealth or traditional occupation and are given reservation by the government of India [32].

Subjective socioeconomic status was assessed with the question, “Please imagine a ten-step ladder, where at the bottom are the people who are the worst off – who have the least money, least education, and the worst jobs or no jobs, and at the top of the ladder are the people who are the best off – those who have the most money, most education, and best jobs. Please indicate the number (1-10) on the rung on the ladder where you would place yourself.” [17]. Steps 1 to 3 on the socioeconomic ladder were defined as low, 4–5 as medium, and 6–10 as high.

*Organizational religiosity* was assessed with the question, “In the past year, how often have you attended religious services (at a temple/mosque/church, etc.)?” Response options were grouped into 1 (low) = not at all, 2 (medium) = 1–3 times a month or 1 or more times a year, and 3(high) = once a week or more than once a week or every day [17].

*Social participation* was measured with 6 items, e.g., “Eat-out-of-house (restaurant/hotel)” [33]. Responses were coded 1 = daily to at least once a month and 0 = rarely/once a year or never, and social participation was defined as at least one activity [33].

### Data analysis

Sampling weights applied to account for both study design (stratification) and non-response. Considering the clustered study design, data analyses were conducted with “STATA software version 15.0 (Stata Corporation, College Station, TX, USA).” Logistic, linear and Poisson regression analyses were used to calculate associations between moderate and high perceived discrimination as well as attribution of discrimination, and binary, scale and count outcome variables. Odds ratios and 95 Confidence Intervals (CI) are presented for logistic regression analyses, exponential Coefficients and 95% CI for linear regression, and Incident Risk Ratios and 95% CI for Poison regression analyses. The first multivariable models (Model 1) were adjusted for age group, and sex, and in the second multivariable models (Model 2) adjustments were made for age group, sex, education, marital status, subjective socioeconomic status, area of residence, caste/tribe, social participation, organised religiosity, and all health indicators assessed in this study. Sociodemographic and social covariates were selected based on a previous literature review [1, 12]. *P*-values of below 0.05 were accepted as significant and missing values were excluded from the analysis.

## Results

### Sample characteristics

The sample included 72,262 middle-aged and older adults (45 years and older), 58.0% were female and 42.0% male. Almost half of the participants (49.5%) had no formal education, 75.6% were married, and 68.2% were residing in rural areas. One in five (24.1%) of the participants rated their socioeconomic status as high, 27.6% attended at least weekly religious services, and 54.4% had social participation. Furthermore, health indictor characteristics are shown in Table 1 (see Table 1).

**Table 1:** Sample characteristics among middle-aged and older adults in India, 2017–2018 (*N* = 72,262)

Variable	Variable specification	Sample	Discrimination	Attribution of discrimination		
		% or M (SD)	≥1 vs 0)	Age	Financial	Other
**Social and demographic factors**						
Age in years	45–59	54.1	17.0	6.3	7.7	7.6
	60 or more	45.9	17.6	10.8	6.4	6.4
Sex	Female	58.0	17.0	8.2	7.2	6.9
	Male	42.0	17.7	8.5	7.0	7.3
Education	≥1 years schooling	50.5	15.1	6.5	5.8	6.4
	No schooling	49.5	19.5	10.2	8.5	7.7
Subjective socioeconomic status	Low	37.2	21.6	10.3	10.4	8.8
	Medium	38.7	15.8	7.0	6.2	6.5
	High	24.1	13.1	7.3	3.6	5.1
Marital status	Not married	24.4	18.9	11.4	7.7	6.4
	Married	75.6	16.8	7.3	6.9	7.2
Caste/tribe	None of the below	24.9	14.0	7.3	5.2	5.4
	Scheduled caste	8.8	19.3	8.8	9.2	8.4
	Scheduled tribe	19.7	16.5	8.3	5.9	7.0
	Other backward class	46.7	18.8	8.9	7.6	7.5
Residence	Rural	68.2	18.1	8.8	7.8	7.0
	Urban	31.8	15.6	7.2	5.6	7.0
Organised religiosity (Attendance of religious services)	Not at all	25.5	15.4	7.8	5.5	6.9
	1–3 times/month or ≥ 1 times/year	46.9	17.2	8.3	7.6	6.9
	≥1/week or every day	27.6	19.1	8.9	7.8	7.4
Social participation	Yes	54.4	19.4	9.1	7.9	8.3
**Mental health**						
Life satisfaction	Low	11.1	29.6	13.6	15.7	12.3
Cognitive functioning	Scale (0–32): M (SD)	18.7 (5.1)	17.8 (5.2)	12.3 (5.3)	17.6 (5.1)	17.8 (5.2)
Insomnia symptoms	Yes	12.7	26.4	13.8	11.3	9.5
Depressive symptoms	Yes	27.6	28.9	14.3	13.3	11.8
**Physical health**						
*Any pain conditions*	1 or more	65.7	19.3	9.5	8.1	7.9
Back pain or problem	Yes	31.5	20.8	10.0	9.1	8.1
Persistent headaches	Yes	12.8	21.9	9.5	11.1	8.6
Painful teeth	Yes	28.4	21.5	10.4	8.7	9.7
Pain or stiffness of joints	Yes	46.1	20.2	10.2	8.6	8.2
*Any cardiovascular conditions*	1 or more	46.6	16.7	8.5	6.8	6.8
Hypertension	Yes	40.4	16.0	8.4	6.3	6.5
Angina	Yes	8.6	19.9	8.6	9.7	7.5
Heart disease	Yes	8.6	14.2	7.6	5.8	5.2
Stoke	Yes	1.8	22.7	13.5	6.7	12.8
*Chronic lung disease*	Yes	6.3	19.7	11.5	8.3	7.3
*Functional limitations*^*a*^	2 or more	28.8	22.1	11.6	8.6	9.3
**Health risk behaviour**						
Current tobacco smoking	Yes	30.4	19.1	9.6	8.3	7.0
Heavy episodic drinking	Yes	2.9	24.8	13.4	10.2	11.5
Physical inactivity	Yes	23.7	20.1	11.2	5.7	7.5

^a^Difficulties with two or more Activities of Daily Living (ADL) and Instrumental Activities of Daily Living (IADL)

### Proportions of perceived discrimination and reasons

The prevalence of moderate (1–2 types) perceived discrimination was 10.7%, and high (3–6 types) perceived discrimination was 6.6%. Among those participants who reported having experienced discrimination, the perceived reasons were age 48.9%, financial status 41.8%, caste 12.9%, gender 9.3%, other 8.9%, other aspects of physical appearance 6.3%, religion 5.3%, physical disability 3.9%, and weight 1.6% (see Table 2).

**Table 2:** Proportions of perceived discrimination and reasons

Perceived discrimination	Treated with less courtesy or respect.	12.1
	Poorer service at restaurants or stores.	6.7
	People act as if they think you are not smart.	8.1
	People act as if they are afraid of you.	5.1
	You are threatened or harassed.	6.4
	Poorer medical services or treatment.	5.9
Number of perceived discriminations	0	82.7
	1–2	10.7
	3–6	6.6
Reasons of discrimination	Age	8.3
	Financial	7.1
	Caste	2.2
	Gender	1.6
	Physical appearance	1.1
	Religion	0.9
	Physical disability	0.7
	Body weight	0.3
	Other	1.5

### Associations of moderate and high perceived discrimination with health outcome indicators

In the final adjusted logistic, linear or Poisson regression analyses, moderate and/or high perceived discrimination was significantly positively associated with poor mental health (low life satisfaction, poor cognitive functioning, insomnia symptoms, and depressive symptoms), pain conditions count, functional limitations, and health risk behaviours (heavy episodic drinking and physical inactivity). High perceived discrimination was in the first model adjusted for age and sex associated with current tobacco use, while no associations were found for cardiovascular conditions count and chronic lung disease (see Table 3).

**Table 3:** Associations between perceived discrimination and health indicators

Outcome variables	Response format	Perceived discrimination	Model 1: adjusted odds ratio or exp. (Coef.) (95% CI)^a^	Model 2: adjusted odds ratio, IRR, or exp. (Coef.) (95% CI)^b^
**Mental health**				
Low life satisfaction	No	No	1 Reference	1 Reference
	Yes	Moderate	2.11 (1.73, 2.58)***	1.47 (1.28, 1.68)***
		Severe	2.48 (2.13, 2.89)***	1.42 (1.20, 1.67)***
Cognitive functioning	Scale	No	1 Reference	1 Reference
		Moderate	0.39 (0.28, 0.54)***	0.60 (0.49, 0.74)***
		Severe	0.29 (0.20, 0.42)***	0.71 (0.55, 0.93)*
Insomnia symptoms	No	No	1 Reference	1 Reference
	Yes	Moderate	1.73 (1.50, 2.00)***	1.45 (1.25, 1.57)***
		Severe	2.15 (1.84, 2.51)***	1.43 (1.22, 1.69)***
Depressive symptoms	No	No	1 Reference	1 Reference
	Yes	Moderate	1.91 (1.64, 2.22)***	1.40 (1.18, 1.65)***
		Severe	5.02 (4.47, 6.62)***	4.14 (3.62, 4.74)***
**Physical health**				
Number of pain conditions	Count	No	1 Reference	1 Reference
		Moderate	1.20 (1.08, 1.32)***	1.06 (1.00, 1.12)*
		Severe	1.36 (1.30, 1.44)***	1.19 (1.13, 1.26)***
Number of cardiovascular conditions	Count	No	1 Reference	1 Reference
		Moderate	0.94 (0.87, 1.01)	0.93 (0.87, 1.00)
		Severe	1.01 (0.94, 1.09)	0.99 (0.92, 1.06)
Chronic lung disease	No	No	1 Reference	1 Reference
	Yes	Moderate	1.24 (0.97, 1.58)	1.21 (0.93, 1.57)
		Severe	1.08 (0.88, 1.31)	1.01 (0.83, 1.23)
Functional limitations^c^	No	No	1 Reference	1 Reference
	Yes	Moderate	1.75 (1.53, 2.01)***	1.55 (1.35, 1.78)***
		Severe	1.44 (1.25, 1.65)***	1.15 (1.00, 1.33)*
**Health risk behaviour**				
Current tobacco smoking	No	No	1 Reference	1 Reference
	Yes	Moderate	1.04 (0.87, 1.26)	1.01 (0.87, 1.16)
		Severe	1.29 (1.11, 1.49)***	1.13 (0.97, 1.33)
Heavy episodic drinking	No	No	1 Reference	1 Reference
	Yes	Moderate	1.29 (0.99, 1.67)	1.31 (1.01, 1.70)*
		Severe	2.05 (1.33, 3.15)***	1.66 (1.25, 2.20)***
Physical inactivity	No	No	1 Reference	1 Reference
	Yes	Moderate	1.06 (0.91, 1.24)	1.06 (0.90, 1.25)
		Severe	1.67 (1.44, 1.94)***	1.61 (1.37, 1.89)***

*CI* Confidence Interval; ***p < 0.001;**p < 0.01; *p < 0.05; ^a^Adjusted for age group, and sex, ^b^Adjusted for age group, sex, education, marital status, subjective socioeconomic status, area of residence, caste/tribe, social participation, organised religiosity, and all other variables in the table; ^c^Difficulties with two or more Activities of Daily Living (ADL) and Instrumental Activities of Daily Living (IADL), *IRR* Incident Risk Ratio

### Associations between reasons of discrimination and health outcomes

In the final adjusted logistic or Poisson regression models, perceived age and/or financial discrimination were positively associated with low life satisfaction, depressive symptoms, pain conditions, and physical inactivity. In addition, caste/tribe was positively associated with functional limitations and depressive symptoms (see Table 4).

**Table 4:** Associations between reasons of discrimination and selected health outcomes

Outcome variables	Attribution of discrimination	Model 1: adjusted odds ratio or IRR (95% CI)^a^	Model 2: adjusted odds ratio or IRR (95% CI)^b^
**Mental health**			
Low life satisfaction	No discrimination	1 Reference	1 Reference
	Age	1.30 (1.09, 1.55)**	1.25 (1.06, 1.47)**
	Financial	2.66 (2.31, 3.06)***	2.33 (2.00, 2.72)***
	Caste	1.43 (1.12, 1.85)**	1.12 (0.85, 1.48)
	Gender	1.06 (0.71, 1.60)	0.94 (0.55, 1.63)
Depressive symptoms	No discrimination	1 Reference	1 Reference
	Age	1.91 (1.70, 2.15)***	1.72 (1.50, 1.96)***
	Financial	2.45 (2.17, 2.76)***	2.07 (1.81, 2.36)***
	Caste	1.59 (1.22, 2.07)***	1.76 (1.43, 2.17)***
	Gender	1.36 (0.90, 2.05)	1.14 (0.67, 1.94)
**Physical health**			
Number of pain conditions	No discrimination	Reference	Reference
	Age	1.12 (1.05, 1.18)***	1.08 (1.02, 1.14)**
	Financial	1.23 (1.16, 1.31)***	1.15 (1.08, 1.23)***
	Caste	1.13 (1.01, 1.27)*	0.98 (0.91, 1.06)
	Gender	1.03 (0.91, 1.16)	1.02 (0.93, 1.12)
Functional limitations^c^	No discrimination	1 Reference	1 Reference
	Age	1.39 (1.20, 1.61)***	1.23 (1.07, 1.43)**
	Financial	1.36 (1.20, 1.55)***	1.21 (1.06, 1.39)**
	Caste	1.12 (1.02, 1.23)*	1.15 (1.04, 1.28)**
	Gender	1.31 (0.74, 2.32)	1.21 (1.06, 1.39)
**Health risk behaviour**			
Physical inactivity	No discrimination	1 Reference	1 Reference
	Age	1.54 (1.33, 1.77)***	1.47 (1.27, 1.69)***
	Financial	0.69 (0.60, 0.80)***	0.68 (0.59, 0.76)***
	Caste	0.98 (0.80, 1.18)	0.91 (0.79, 1.03)
	Gender	0.67 (0.43, 1.05)	0.68 (0.44, 1.07)

*CI* Confidence Interval; ***p < 0.001;**p < 0.01; *p < 0.05; ^a^Adjusted for age group, and sex, ^b^Adjusted for age group, sex, education, marital status, subjective socioeconomic status, area of residence, caste/tribe, social participation, organised religiosity, and all health variables; ^c^Difficulties with two or more Activities of Daily Living (ADL) and Instrumental Activities of Daily Living (IADL), *IRR* Incident Risk Ratio

## Discussion

The study aimed to estimate the associations between perceived discrimination, poor physical health, poor mental health, and health risk behaviours in middle-aged and older adults in a national population survey in India in 2017–2018. Results show for the first time that perceived discrimination is associated with poor mental health (low life satisfaction, poor cognitive functioning, insomnia symptoms, and depressive symptoms), poor physical health (pain conditions, and functional limitations), and health risk behaviours (heavy episodic drinking and physical inactivity) in middle-aged and older adults in India. This was found after adjusting for relevant confounders, including age group, sex, education, marital status, subjective socioeconomic status, area of residence, social participation, organised religiosity, and all health variables assessed in this study. These results are consistent with previous research [1, 4–12] in high-income countries. Generally, perceived discrimination associations were stronger on mental than physical health, which concurs with previous observations [7].

The association between perceived discrimination with poor mental health (low life satisfaction, poor cognitive functioning, insomnia symptoms, and depressive symptoms), is possibly confirming a direct pathway from increases in perceived discrimination to more negative mental health outcomes, as found in predominantly high-income countries [1]. Everyday discrimination may act as micro-aggressors believed to lead to defensive anxiety and a state of chronic stress, leading to poorer mental health over time [10]. In addition, persons with perceived discrimination may activate a stereotype thread, in particular age-based stereotype threat, which was found among older adults to have led to decreased performance in cognition tests [10, 34].

Possible mechanisms relate to perceived discrimination as stressors that are uncontrollable and unpredictable and thus detrimental to health, initiating a process of physiological responses, such as increased cortisol secretion, heart rate, blood pressure, chronic pain, respiratory problems, and functional limitations which overtime negatively impact health [1, 10, 12, 31]. However, in our study we only found associations between perceived discrimination and pain condition counts and functional limitations, but not with cardiovascular condition count and respiratory problems or chronic lung disease, as found in some previous studies [4, 35].

Regarding health risk behaviours, this study found an association between perceived discrimination and heavy episodic drinking, physical inactivity, and in the not fully adjusted analysis tobacco smoking. Perceived discrimination may reduce self-control capabilities, potentially increasing different types of health risk behaviours [1]. For example, individuals with perceived discrimination may cope with this stressor by “self-medication” with heavy drinking and/or using nicotine [9]. In addition, “It is possible that people who use tobacco and/or alcohol products do so to strengthen social relationships with other substance users.” [36].

Among nine attributions of discrimination, the highest were age and financial, with a lower rate of caste and gender discrimination and the lowest physical disability and body weight discrimination, and age and financial discrimination were associated with almost all health outcomes in this study. In comparison, in USA, the four most common reasons for perceived discrimination were race-ethnicity, gender, various aspects of appearance (predominantly weight), and age [37], and in European countries [38], the highest was age discrimination, followed by gender and ethnic discrimination. Our results seem to mean that discrimination primarily affects the age and materially disadvantaged population in India. A previous study among older adults India showed that lower socioeconomic status (subjective socioeconomic status, food insecurity, and no education) was associated with discrimination experiences [39], may be because of higher exposure to discriminatory situations [40], and belonging to lower castes (scheduled tribes and other backward classes) [14]. Moreover, studies have shown that economic, caste and gender inequality negatively impacts on health outcomes [41], self-reported morbidity and untreated morbidity [42], and violence [43] among adults and older adults in India, emphasizing the need for “social and economic support should be given to the economically vulnerable older population.” [43]. In contrast, in many American studies race-based chronic discrimination (both actual and perceived) is the focus setting into “motion, a process of physiological responses (e.g., elevated blood pressure and heart rate, production of biochemical reactions, hypervigilance) that eventually result in disease and mortality.” [44].

### Strength and limitations of the study

Study strengths included the use of standardized measures adapted from the Health and Retirement Study and a large, nationally representative sample of middle-aged and older adults in India. The self-report of most data may have their limitations. It is possible that participants underreported everyday discrimination experiences, and some chronic conditions were not verified by clinical diagnosis. Although these events are not objective observed discrimination, they can be considered as a form of stress [1]. Due to the cross-sectional design of the study, we were unable to determine the direction of the assessed associations. Some variables, such as dietary behaviour, were not assessed and should be included in future studies. A further limitation was that the role of structural discrimination was not assessed. It is likely that people who experience everyday discrimination are also experiencing structural discrimination. Another study limitation was that we have no information about whether the study sample has good representation of groups of people who are historically under-represented in research studies, such as those of lower casts or socioeconomic status groups.

## Conclusions

The study largely extents existing evidence from high-income countries that have shown associations between perceived discrimination and low life satisfaction, poor cognitive functioning, insomnia symptoms, depressive symptoms, pain conditions, functional limitations, heavy episodic drinking and physical inactivity among middle-aged and older adults in India. It would be important to address perceived discrimination in Indian society, and by using such measures, harmful effects of discrimination on mental health, cognitive functioning, pain conditions, functional limitations, and health risk behaviours could be reduced at an early stage.

## Data Availability

The data are available at the The Gateway to Global Aging Data (www.g2aging.org).

## Abbreviations

ADL: Activities of Daily Living
BMI: Body Mass Index
CES-D: Center for Epidemiologic Studies Depression Scale
EDS: Everyday Discrimination Scale
IADL: Instrumental Activities of Daily Living
LASI: Longitudinal Aging Study in India

## Declarations

All methods were carried out in accordance with relevant guidelines and regulations.
